# Dimethyl 4,5-dichlorobenzene-1,2-dicarboxyl­ate

**DOI:** 10.1107/S1600536812007167

**Published:** 2012-02-24

**Authors:** Yongling Sun

**Affiliations:** a Department of Biology, Dezhou University, Dezhou 253023, People’s Republic of China

## Abstract

In the title compound, C_10_H_8_Cl_2_O_4_, the two Cl atoms and one of the meth­oxy­carbonyl groups are almost coplanar [maxi­mum derivation = 0.035 (2) Å] with the benzene plane, and the other meth­oxy­carbonyl group exhibits an almost orthogonal disposition relative to the benzene plane, with a dihedral angle of 84.82 (3)° between the planes. In the crystal, the molecules are connected into a chain propagating along the [011] direction through nonclassical C—H⋯O hydrogen bonds.

## Related literature

For the chemical properties and structural nature nature of some related benzene­carboxyl­ate derivatives, see: Galešić *et al.* (1984 ▶); Liang *et al.* (2004 ▶); Mallinson *et al.* (2003 ▶); Rauf *et al.* (2008 ▶).
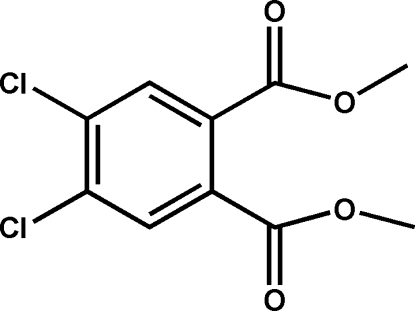


## Experimental

### 

#### Crystal data

C_10_H_8_Cl_2_O_4_
*M* *_r_* = 263.06
Triclinic, 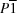

*a* = 7.1906 (14) Å
*b* = 7.8410 (17) Å
*c* = 10.6205 (15) Å
α = 97.779 (15)°
β = 109.040 (15)°
γ = 91.864 (18)°
*V* = 558.95 (19) Å^3^
*Z* = 2
Mo *K*α radiation
μ = 0.58 mm^−1^
*T* = 295 K
0.24 × 0.20 × 0.18 mm

#### Data collection

Bruker SMART 1000 CCD diffractometer
Absorption correction: multi-scan (*SADABS*; Sheldrick, 1996 ▶) *T*_min_ = 0.871, *T*_max_ = 0.902
3362 measured reflections
1966 independent reflections
1663 reflections with *I* > 2σ(*I*)
*R*_int_ = 0.010

#### Refinement

*R*[*F* ^2^ > 2σ(*F* ^2^)] = 0.035
*wR*(*F* ^2^) = 0.092
*S* = 1.03
1966 reflections
147 parameters
H-atom parameters constrained
Δρ_max_ = 0.28 e Å^−3^
Δρ_min_ = −0.24 e Å^−3^

### 

Data collection: *SMART* (Siemens, 1996 ▶); cell refinement: *SAINT* (Siemens, 1996 ▶); data reduction: *SAINT*; program(s) used to solve structure: *SHELXS97* (Sheldrick, 2008 ▶); program(s) used to refine structure: *SHELXL97* (Sheldrick, 2008 ▶); molecular graphics: *SHELXTL* (Sheldrick, 2008 ▶); software used to prepare material for publication: *SHELXTL*.

## Supplementary Material

Crystal structure: contains datablock(s) I, global. DOI: 10.1107/S1600536812007167/rk2334sup1.cif

Structure factors: contains datablock(s) I. DOI: 10.1107/S1600536812007167/rk2334Isup2.hkl

Supplementary material file. DOI: 10.1107/S1600536812007167/rk2334Isup3.cml

Additional supplementary materials:  crystallographic information; 3D view; checkCIF report

## Figures and Tables

**Table 1:** Hydrogen-bond geometry (Å, °)

*D*—H⋯*A*	*D*—H	H⋯*A*	*D*⋯*A*	*D*—H⋯*A*
C6—H6⋯O4^i^	0.93	2.37	3.278 (2)	164

Symmetry code: (i) 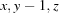.

**Fig. 1.**
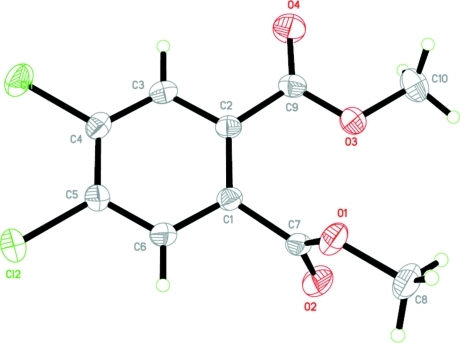
The molecular structure of the title compound with the atom numbering scheme. Displacement ellipsoids are drawn at 30% probability level. H atoms are presented as a small spheres of arbitrary radius.

## supplementary crystallographic information

### Comment

Benzenecarboxylate derivatives have been extensively studied due to their excellent chemical properties and easily modified structural natures. (Mallinson *et al.*, 2003 and Liang *et al.*, 2004). Furthermore, the investigate on single-crystal structure of benzenecarboxylate derivatives have become increasingly important in revealing precisely the relation between their chemical properties and molecular structures (Galešić *et al.*, 1984 and Rauf *et al.*, 2008). As an extension of our work on benzenecarboxylate structural characterization, the title compound, I, is synthesized and characterized by X-ray diffraction, as shown in Fig. 1.

The compound I crystallizes in the triclinic system and consists of one phenyl framework, together with two chlorine atoms and two methoxycarbonyl groups linked to its peripheral position, respectively. Two chlorine atoms are co-planar with the benzene ring, companying the maximum deviation of 0.035 (2)Å from this benzene plane. Furthermore, one methoxycarbonyl group is also co-planar with this benzene plane, with the dihedral angel of 2.03 (3)° between the methoxycarbonyl plane of C9–O3–O4–C10 and the benzene plane. In contrast, the other methoxycarbonyl plane of C7–O1–O2–C8 exhibits almostly orthogonal configuration in relative to this benzene plane with the dihedral angle of 84.82 (3)° between them. As shown in Table 1, the distances of C–O (esterified hydroxyl oxygen atom) locating in the range of 1.321 (2)-1.448 (2)Å, clearly indicate their typical single-bond nature in contrast to the obviously double-bond of C═O (carbonyl oxygen atom) 1.187 (2)Å and 1.193 (2)Å, revealing the excellent flexible bridge nature of these methoxycarbonyl moieties. Furthermore, this compound molecules are connected into one-dimension chain along the [0 1 1] direction through H bond C6–H6···O4^i^ with H6···O4^i^ distance of 2.373 (3)°. Symmetry code: (i) *x*, *y*-1, *z*.

### Experimental

To the solution of 4,5-dichloro-1,2-benzenedicarboxyl acid (466 mg, 2 mmol) in *Me*OH (50 ml), one drop of H_2_SO_4_ was added. After refluxed for five hours under N_2_ atmosphere, the resulting mixture was evaporated, and the residue was chromatographed on a silica gel column using CHCl_3_ as eluent. Repeated chromatography followed by recrystallization from CHCl_3_ and *Me*OH gave the target compound as white crystals. Yield: 182 mg, 34.6%. Anal. for C_10_H_8_Cl_2_O_4_: Calc. C, 45.66; H, 3.07; Found: C, 45.42; H, 3.17. The No. of CCDC: 863226.

### Refinement

All H atoms were placed in geometrically idealized positions and treated as riding on their parent atoms with C–H distances of 0.93Å with *U*_iso_(H) = 1.2*U*_eq_(C) for aryl H atoms and C–H distances of 0.96Å with *U*_iso_(H) = 1.5*U*_eq_(C) for methyl H atoms. The CCDC deposit number 863226.

### Crystal data

**Table:**

C_10_H_8_Cl_2_O_4_	*Z* = 2
*M**_r_* = 263.06	*F*(000) = 268
Triclinic, *P*1	*D*_x_ = 1.563 Mg m^−^^3^
Hall symbol: -P 1	Melting point: 389 K
*a* = 7.1906 (14) Å	Mo *K*α radiation, λ = 0.71073 Å
*b* = 7.8410 (17) Å	Cell parameters from 1973 reflections
*c* = 10.6205 (15) Å	θ = 2.4–25.0°
α = 97.779 (15)°	µ = 0.58 mm^−^^1^
β = 109.040 (15)°	*T* = 295 K
γ = 91.864 (18)°	Block, colourless
*V* = 558.95 (19) Å^3^	0.24 × 0.20 × 0.18 mm

### Data collection

**Table:**

Bruker SMART 1000 CCD diffractometer	1966 independent reflections
Radiation source: fine-focus sealed tube	1663 reflections with *I* > 2σ(*I*)
Graphite monochromator	*R*_int_ = 0.010
φ and ω scans	θ_max_ = 25.0°, θ_min_ = 3.1°
Absorption correction: multi-scan (*SADABS*; Sheldrick, 1996)	*h* = −7→8
*T*_min_ = 0.871, *T*_max_ = 0.902	*k* = −9→8
3362 measured reflections	*l* = −12→12

### Refinement

**Table:**

Refinement on *F*^2^	Primary atom site location: structure-invariant direct methods
Least-squares matrix: full	Secondary atom site location: difference Fourier map
*R*[*F*^2^ > 2σ(*F*^2^)] = 0.035	Hydrogen site location: inferred from neighbouring sites
*wR*(*F*^2^) = 0.092	H-atom parameters constrained
*S* = 1.03	*w* = 1/[σ^2^(*F*_o_^2^) + (0.0449*P*)^2^ + 0.1781*P*] where *P* = (*F*_o_^2^ + 2*F*_c_^2^)/3
1966 reflections	(Δ/σ)_max_ < 0.001
147 parameters	Δρ_max_ = 0.28 e Å^−^^3^
0 restraints	Δρ_min_ = −0.24 e Å^−^^3^

### Special details

**Table:**

Geometry. All s.u.'s (except the s.u. in the dihedral angle between two l.s. planes) are estimated using the full covariance matrix. The cell s.u.'s are taken into account individually in the estimation of s.u.'s in distances, angles and torsion angles; correlations between s.u.'s in cell parameters are only used when they are defined by crystal symmetry. An approximate (isotropic) treatment of cell s.u.'s is used for estimating s.u.'s involving l.s. planes.
Refinement. Refinement of *F*^2^ against ALL reflections. The weighted *R*-factor *wR* and goodness of fit *S* are based on *F*^2^, conventional *R*-factors *R* are based on *F*, with *F* set to zero for negative *F*^2^. The threshold expression of *F*^2^ > σ(*F*^2^) is used only for calculating *R*-factors(gt) *etc*. and is not relevant to the choice of reflections for refinement. *R*-factors based on *F*^2^ are statistically about twice as large as those based on *F*, and *R*-factors based on ALL data will be even larger.

### Fractional atomic coordinates and isotropic or equivalent isotropic displacement parameters (Å^2^)

**Table:**

	*x*	*y*	*z*	*U*_iso_*/*U*_eq_	
Cl1	0.31371 (9)	0.97739 (8)	0.82726 (5)	0.0629 (2)	
Cl2	0.25088 (11)	0.57862 (8)	0.71088 (6)	0.0693 (2)	
O1	0.3423 (2)	0.70631 (19)	0.21566 (13)	0.0517 (4)	
O3	0.2024 (2)	1.05587 (17)	0.22604 (14)	0.0524 (4)	
C7	0.1745 (3)	0.7231 (2)	0.24061 (19)	0.0404 (4)	
C9	0.2309 (3)	1.1128 (2)	0.3543 (2)	0.0414 (4)	
C1	0.2123 (3)	0.7964 (2)	0.38630 (18)	0.0365 (4)	
C6	0.2187 (3)	0.6771 (2)	0.47290 (19)	0.0449 (5)	
H6	0.2027	0.5596	0.4397	0.054*	
C3	0.2674 (3)	1.0250 (2)	0.57299 (19)	0.0401 (4)	
H7	0.2835	1.1423	0.6069	0.048*	
C2	0.2367 (2)	0.9725 (2)	0.43690 (18)	0.0349 (4)	
C4	0.2743 (3)	0.9063 (3)	0.65837 (19)	0.0404 (4)	
C5	0.2487 (3)	0.7316 (2)	0.60792 (19)	0.0431 (5)	
O2	0.0140 (2)	0.6798 (2)	0.16088 (15)	0.0648 (4)	
O4	0.2507 (4)	1.26184 (19)	0.39955 (19)	0.0862 (6)	
C10	0.1877 (4)	1.1857 (3)	0.1392 (3)	0.0655 (7)	
H13A	0.1488	1.1307	0.0470	0.098*	
H13C	0.3134	1.2499	0.1630	0.098*	
H13B	0.0910	1.2628	0.1499	0.098*	
C8	0.3252 (4)	0.6420 (3)	0.0777 (2)	0.0654 (7)	
H14C	0.2624	0.7232	0.0206	0.098*	
H14A	0.2476	0.5331	0.0491	0.098*	
H14B	0.4544	0.6271	0.0720	0.098*	

### Atomic displacement parameters (Å^2^)

**Table:**

	*U*^11^	*U*^22^	*U*^33^	*U*^12^	*U*^13^	*U*^23^
Cl1	0.0775 (4)	0.0733 (4)	0.0381 (3)	−0.0010 (3)	0.0256 (3)	−0.0048 (3)
Cl2	0.1004 (5)	0.0595 (4)	0.0460 (3)	−0.0028 (3)	0.0175 (3)	0.0212 (3)
O1	0.0606 (10)	0.0574 (9)	0.0324 (7)	0.0079 (7)	0.0121 (7)	−0.0017 (6)
O3	0.0733 (10)	0.0391 (8)	0.0445 (8)	0.0031 (7)	0.0171 (7)	0.0124 (6)
C7	0.0532 (12)	0.0281 (9)	0.0347 (10)	−0.0003 (8)	0.0077 (9)	0.0049 (7)
C9	0.0407 (11)	0.0328 (10)	0.0472 (11)	0.0020 (8)	0.0104 (9)	0.0056 (8)
C1	0.0370 (10)	0.0335 (9)	0.0339 (10)	0.0009 (7)	0.0062 (8)	0.0027 (7)
C6	0.0580 (13)	0.0320 (10)	0.0392 (11)	−0.0003 (8)	0.0104 (9)	0.0028 (8)
C3	0.0380 (10)	0.0352 (10)	0.0434 (11)	0.0014 (8)	0.0124 (8)	−0.0028 (8)
C2	0.0299 (9)	0.0330 (9)	0.0388 (10)	0.0016 (7)	0.0083 (8)	0.0034 (7)
C4	0.0346 (10)	0.0500 (11)	0.0343 (10)	0.0003 (8)	0.0112 (8)	0.0007 (8)
C5	0.0474 (11)	0.0428 (11)	0.0373 (10)	0.0000 (8)	0.0109 (9)	0.0089 (8)
O2	0.0618 (10)	0.0745 (11)	0.0412 (9)	−0.0148 (8)	0.0007 (8)	−0.0018 (8)
O4	0.159 (2)	0.0288 (8)	0.0697 (12)	0.0046 (9)	0.0378 (12)	0.0052 (8)
C10	0.0815 (17)	0.0585 (14)	0.0626 (15)	0.0099 (12)	0.0233 (13)	0.0313 (12)
C8	0.0928 (19)	0.0670 (15)	0.0366 (11)	0.0110 (13)	0.0243 (12)	0.0006 (10)

### Geometric parameters (Å, º)

**Table:**

Cl1—C4	1.7308 (19)	C6—C5	1.383 (3)
Cl2—C5	1.7260 (19)	C6—H6	0.9300
O1—C7	1.324 (2)	C3—C4	1.376 (3)
O1—C8	1.447 (2)	C3—C2	1.390 (3)
O3—C9	1.321 (2)	C3—H7	0.9300
O3—C10	1.448 (2)	C4—C5	1.385 (3)
C7—O2	1.193 (2)	C10—H13A	0.9600
C7—C1	1.508 (3)	C10—H13C	0.9600
C9—O4	1.187 (2)	C10—H13B	0.9600
C9—C2	1.490 (3)	C8—H14C	0.9600
C1—C6	1.390 (3)	C8—H14A	0.9600
C1—C2	1.396 (3)	C8—H14B	0.9600
			
C7—O1—C8	116.24 (17)	C1—C2—C9	124.47 (17)
C9—O3—C10	116.45 (16)	C3—C4—C5	119.57 (17)
O2—C7—O1	125.13 (18)	C3—C4—Cl1	119.55 (15)
O2—C7—C1	123.77 (19)	C5—C4—Cl1	120.87 (15)
O1—C7—C1	111.03 (16)	C6—C5—C4	120.08 (17)
O4—C9—O3	123.07 (18)	C6—C5—Cl2	118.90 (15)
O4—C9—C2	123.22 (19)	C4—C5—Cl2	121.02 (15)
O3—C9—C2	113.71 (15)	O3—C10—H13A	109.5
C6—C1—C2	119.35 (17)	O3—C10—H13C	109.5
C6—C1—C7	116.17 (16)	H13A—C10—H13C	109.5
C2—C1—C7	124.47 (16)	O3—C10—H13B	109.5
C5—C6—C1	120.58 (17)	H13A—C10—H13B	109.5
C5—C6—H6	119.7	H13C—C10—H13B	109.5
C1—C6—H6	119.7	O1—C8—H14C	109.5
C4—C3—C2	121.09 (17)	O1—C8—H14A	109.5
C4—C3—H7	119.5	H14C—C8—H14A	109.5
C2—C3—H7	119.5	O1—C8—H14B	109.5
C3—C2—C1	119.31 (17)	H14C—C8—H14B	109.5
C3—C2—C9	116.21 (16)	H14A—C8—H14B	109.5

### Hydrogen-bond geometry (Å, º)

**Table:**

*D*—H···*A*	*D*—H	H···*A*	*D*···*A*	*D*—H···*A*
C6—H6···O4^i^	0.93	2.37	3.278 (2)	164

Symmetry code: (i) *x*, *y*−1, *z*.
